# Crosstalk between autophagy and apoptosis induced by camphor in Schizosaccharomyces pombe

**DOI:** 10.3906/biy-1908-11

**Published:** 2019-12-13

**Authors:** Hızlan Hıncal AĞUŞ, Sedanur YILMAZ, Cansın Ogeday ŞENGÖZ

**Affiliations:** 1 Department of Molecular Biology and Genetics, Faculty of Arts & Science, İstanbul Yeni Yüzyıl University, İstanbul Turkey

**Keywords:** Camphor, autophagy, apoptosis, Atg8, Aif1, Schizosaccharomyces pombe

## Abstract

Camphor is widely used in pharmacy, the food industry, and cosmetics. In this study, we evaluate inhibitory and cytotoxic effects of camphor in the fission yeast (*Schizosaccharomyces pombe*), which presents a unicellular model in mechanistic toxicology and cell biology. Low-dose camphor exposure (0.4 mg/mL) activated autophagy, which was shown by GFP-Atg8 dots and transcriptional upregulation of Atg6 (Beclin-1 ortholog). Autophagy was also confirmed by using autophagy-deficient cells, which showed reduction in GFP-Atg8 dot formation. However, high-dose camphor exposure (0.8 mg/mL) caused dramatic cell death ratios, demonstrated by spot and colony-forming assays, even in autophagy-deficient cells. To unravel the underlying mechanism, this time, apoptosis-deficient cells were exposed to low- and high-dose camphor. Apoptosis was also confirmed by acridine orange/ethidium bromide staining. Among yeast apoptosis mediators, Aif1 was found to mediate camphor-induced cell death. In conclusion, differential regulation of autophagy and apoptosis, and switches between them, were found to be dose-dependent. The potential effects of camphor on autophagy and apoptotic cell death and underlying mechanisms were clarified in basic unicellular eukaryotic model, *S. pombe*.

## 1. Introduction

Terpenes and terpenoids are herbal extracts extracted from resins of medicinal and aromatic plants including *Citrus*, Lamiaceae, and Pinaceae, or produced from marine organisms such as *Spongia *(Gershenzon and Dudareva, 2007). Many terpenes are defined as harmful chemicals having prooxidant, cytotoxic, genotoxic, and neurotoxic effects (Chueca et al., 2014; Sharma et al., 2019), whereas some other studies (Bastaki et al., 2018; Askari and Shafiee-Nick, 2019) showed antioxidant, antiinflammatory, and cytoprotective effects. Camphor (1,7,7-trimethylbicyclo[2.2.1]heptan-2-one), which is classified among the bicyclic monoterpenes constituted of 2 isoprene units and 10 carbon atoms, is a white-colored powder and found in the camphor tree and other aromatic and medicinal plants (Frizzo et al., 2000).

Camphor was reported to show cytotoxicity characterized by oxidative stress, mitochondrial disruption, apoptosis and/or necrosis, and suppression of cell proliferation at high concentrations (>50 µg/L) in rat thymocytes (Cherneva et al., 2012), human colon cancer cell lines (Itani et al., 2008), and fungi (Agus et al., 2019). In addition, glutathione S-transferase, cytochrome b5, and aryl hydrocarbon hydroxylase activities were increased in response to a 300 mg/kg camphor/body weight dose in mouse liver (Banerjee et al., 1995). Although the dose-dependent and tissue-specific cytotoxic and/or cytoprotective effects of camphor were defined in different organisms and various types of mammalian cell lines (Jeon et al., 2014; Sedaghat and Torshizi, 2017; Sokolova et al., 2018), potential anticancer activities, together with mechanisms of action, are under debate and should be unraveled.

The characteristics of fission yeast (*Schizosaccharomyces pombe*) that are analogous to mammals (Lin and Austriaco, 2014; Koyama et al., 2017, including mitochondrial biogenesis, cell cycle control, and evolutionarily conserved programmed/regulated cell death, in addition to a small and easily manipulated genome (Wood et al., 2002), make this yeast species an excellent model organism for molecular biology, biochemistry, and genetics studies (Hagan et al., 2016; Gerganova et al., 2019). Besides, fission yeast provides a valuable opportunity to be used in cancer research, for its high proliferation rate resembles the Crabtree and Warburg effect (reprogrammed energy metabolism of cancer cells) (Flores et al., 2000; Carmona-Gutierrez et al., 2011; Olayanju et al., 2015). Hence, the cytotoxicity of potential anticancer drugs, along with cell death mechanisms, can be understood using yeast biology (Almeida et al., 2008; Takeda et al., 2011; Villahermosa et al., 2017; Kavakçıoğlu and Tarhan, 2018). 

Camphor toxicity and the underlying mechanisms in model fungi are currently limited, although we have recently reported camphor-induced intrinsic apoptosis due to oxidative stress, mitochondrial impairment, and DNA damage at high doses (0.8–1.2 mg/mL) (Agus et al., 2019). However, camphor-induced autophagy-related growth inhibition or cell death at lower doses was not assessed as a possible inhibition mechanism and should be investigated. Therefore, the fission yeast, as a unicellular eukaryotic model, was used to shed light on the cytotoxicity of camphor and arsenic in this study. To show the autophagy potential of low-dose camphor and the potential roles of autophagy proteins (Atg6, Atg8, Atg9, and Atg14), we used *atg*-deficient and GFP-tagged recombinant cells (*gfp-atg8*). In addition, to distinguish “camphor-induced autophagy-related inhibition” from “apoptotic cell death,” we also used apoptosis-deficient cells (*pca1∆*, *aif1∆*, and *pnu1∆*). We hypothesized that low-dose camphor could cause autophagy without apoptotic cell death, in contrast to camphor-induced apoptosis at higher doses, indicating a cellular decision between autophagy and apoptosis depending on the severity of camphor cytotoxicity. Our data contribute to understanding the inhibitory effects of camphor and further studies are warranted in medicine, pharmacy, and biochemistry.

## 2. Materials and methods

### 2.1. Reagents

Methylene blue was from Merck (İstanbul, Turkey). Components of culture media were from BD Difco (Fisher Scientific, Turkey). Glucose was from Emboy (İstanbul, Turkey). Camphor (product number: 148075-100G) (1,7,7-trimethylbicyclo[2.2.1]heptan-2-one) and arsenic(III) oxide were purchased from Sigma (İstanbul, Turkey).

### 2.2. Yeast strains, media, and growth conditions

*S. pombe* wild-type strain *ED666* (*h- ade6-M210/ura4-D18 leu1-32*), autophagy-deficient cells (*ED666* with *atg6*∆::*KanR*,* atg8*∆*::KanR*,* atg9*∆*::KanR*,* atg14*∆*::KanR*), and**apoptosis-deficient cells**(*ED666 *with* pca1∆::KanR*,* aif1∆::KanR*,**and* pnu1∆::KanR*) were a kind gift from B. Palabıyık (İstanbul University). Cells carrying GFP-tagged Atg8 with the *nmt41* promoter (*SHMT80 h- lys1+::Pnmt41-GFP-atg8+*) were purchased from the NBRP Yeast Genetic Resource Center, Osaka City University. Yeast was grown in standard YEL medium (1% yeast extract, 2% glucose) or EMM media with supplements adenine, leucine, and uracil with or without thiamine. Cells were incubated on a rotary shaker at 180 rpm at 30 °C in all of the experiments. Cultures of 1 × 106 cells/mL from overnight incubation (14 h) were used for experiments.

### 2.3. Camphor exposure, viability, and cytotoxicity

Yeast from overnight culture (OD600 ≈ 1) in YEL media was counted under an optical microscope (Carl-Zeiss, Axio Observer 3) and dispensed to conical flasks at a final concentration of 1 × 106 cells/mL. Camphor was dissolved in ethanol (100 g/L). Cells were exposed to an ethanol control (0.05%) and a graded concentration of camphor (0, 0.4, and 0.8 mg/mL) for 24 h. Arsenic(III) oxide was used as a positive control for testing autophagy and apoptosis (Du et al., 2007). Cell viability was assessed by colony-forming assay. After 1:10 dilution in PBS, 100 µL of cell suspension was spread on a YEA plate and incubated at 30 °C for 3 days. Viability was calculated as the ratio of the experimental group to the control group. Spot assay was performed as previously described (Agus et al., 2019). The YEA medium was prepared with gradually increasing concentrations of camphor. Serial 10-fold dilutions of logarithmic yeast cells were spotted on agar plates with 3 technical replicas and incubated at 30 °C for 3 days.

### 2.4. Total RNA extraction and RT-PCR

After washing 2 times with PBS, total RNA was isolated as previously described (Bähler and Wise, 2017) and DNase I treatment was performed. RNA quantity was evaluated spectrophotometrically at 260 nm and 280 nm; 260/280 ratios were 1.8–2. RNA integrity was determined by 1% agarose gel electrophoresis. A Viva cDNA synthesis kit (Diagen, Turkey) was used for cDNA synthesis. One microgram of total RNA, 1 µM oligo (dT) primer, 1 mM dNTP mix, 1X RT-reaction buffer, and 10 U reverse transcriptase were used for each reaction. Final volume was completed to 20 µLwith molecular-grade water. RT reaction was performed at 42 °C for 1 h. GenTaq PCR mix (GeneMark Biolab) and a Labcycler Thermal Cycler (Sensoquest) were used for PCR reactions. Gene-specific primers were ordered from Sentromer Inc. (Turkey). Primers were used at 0.3 µM quantitation and are presented in the Table. The fold changes were normalized against the *β-actin* reference gene (primer set is given in the Table). PCR reactions for each sample were set as 2 technical replicates and performed in 20 µL total reaction volume. Reaction mix was prepared following the manufacturer’s instructions (GeneMark Biolab, Turkey). Reaction conditions were as follows: incubation at 94 °C for 10 min and 30 cycles of 94 °C for 15 s, 56 °C for 30 s, and 72 °C for 30 s. ImageJ 1.46r Analysis Software (National Institutes of Health, USA) was used to calculate relative band intensities measured against reference gene *Act1*. Mean ± SEM data are calculated from 3 independent biological replicates (n = 3).

### 2.5. Microscopic observation of GFP dots

The yeast-carrying GFP-Atg8 construct was grown in EMM (supplied with uracil, leucine, and adenine) with or without thiamine for regulation of GFP-Atg8 expression under the control of the *nmt41* promoter. After camphor and arsenic(III) oxide exposure, cells were washed 2 times with PBS. Cells were visualized by fluorescence microscopy (Carl-Zeiss, Axio Observer 3) using 63× objectives at λex = 505 nm and λem = 534 nm, and GFP dots were counted within at least 200 cells for each independent biological replicate (n = 3).

### 2.6. Acridine orange/ethidium bromide (AO/EB) dual assay

The acridine orange/ethidium bromide dual staining assay was used to detect apoptosis. Acridine orange, a membrane-permeable dye that can stain cell nuclei, is capable of diffusing through live or dead cell membranes. On the other hand, ethidium bromide can only pass through pores of dead cell membranes and stain the cell nucleus. When acridine orange and ethidium bromide are used at the same time, fragmented bright red-orange cell nuclei will indicate (dead) late apoptotic cells, whereas live cells are only stained by acridine orange and seen as bright-green cells. This experiment was performed as indicated previously (Pajaniradje et al., 2014; Agus et al., 2018). Briefly, cells were mixed with 5 µL of acridine orange/ethidium bromide solution (60 µg/mL of AO:100 µg/mL of EB, dissolved in PBS). After incubation, cells were washed with PBS and examined under a fluorescence microscope (Carl-Zeiss, Axio Observer 3) using 40× objectives at λex = 500 nm and λem = 530 nm for acridine orange and λex = 510 nm and λem = 595 nm for ethidium bromide.

### 2.7. Statistical analysis

The data were expressed as mean ± standard error of the mean (SEM). Differences between groups were analyzed by one-way ANOVA with Tukey’s multiple comparison test using GraphPad Prism (USA). 

## 3. Results

### 3.1. Camphor strongly induces Atg8 activation and autophagosome formation

**Figure 1:**
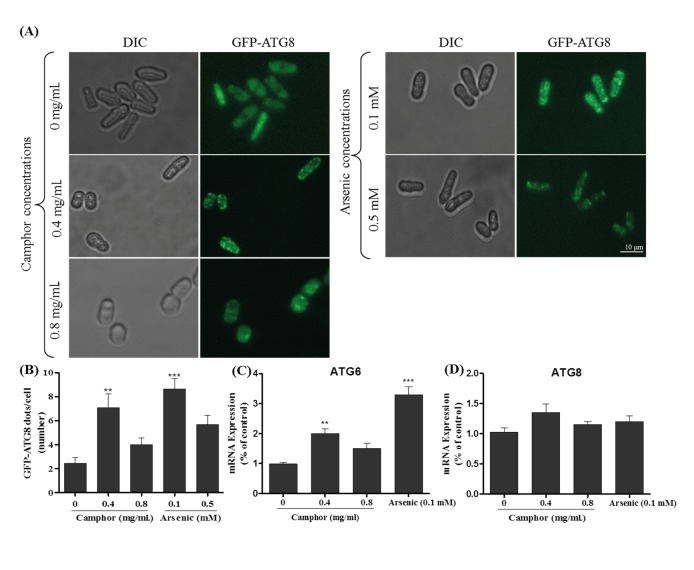
Atg8 activation of S. pombe cells evaluated using GFP-Atg8 recombinant cells. Small GFP dots, which are surrounding autophagosomes, were visualized and counted using fluorescent microscope (using 63× objectives at λex = 505 nm and λem = 534 nm) after exposure to 0, 0.4, and 0.8 mg/mL camphor and 0.1 and 0.5 mM arsenic(III) solutions for 24 h on a rotary shaker at 30 °C and 180 rpm (A). Cells were washed with PBS 2 times. Percentages (B) of GFP-Atg8 dots formed in each cell were calculated by visualizing at least 200 cells in each biological replica (n = 3). GFP dots show autophagosome formation, which are expressed in GFP-Atg8 recombinant cells under the control of the nmt41 promoter. mRNA levels of Atg6 (C) and Atg8 (D) calculated using RT-PCR assay in wild-type cells after exposure to 0–0.8 mg/mL camphor and 0.1–0.5 mM arsenic solutions are shown in corresponding graphics. The fold changes were normalized against β-actin reference gene. Values are presented as mean ± SEM. At least 200 cells were counted in each biological replicate (n = 3).

Atg8 (LC3 ortholog in yeast), an autophagosome membrane binding protein, activates autophagosome formation after proteolytic activation by a specific protease, Atg4. A green signal produced by N-terminal GFP-tagged Atg8 can be visualized using fluorescent microscopy. As an end-point method, small GFP dots, which are surrounding autophagosomes, are counted and compared to that of the untreated group to understand whether Atg8 activation is induced in the experimental group (Torggler et al., 2017). As shown in Figure 1A, GFP dots were visualized using fluorescent microscopy in the presence of camphor and arsenic. Activated and condensed Atg8 (green dots) were counted and average values were calculated as GFP-Atg8 dots/cell as demonstrated in Figure 1B. Exposure to 0.4 mg/mL of camphor significantly increased the number of GFP dots (P**< 0.01); however, this effect was removed when the camphor dose increased twofold (0.8 mg/mL). Low-dose arsenic exposure, as a positive control, caused a dramatic increase in GFP dots/cell ratio (P**< 0.001), whereas there was a mild effect in the high-dose (0.5 mM) arsenic group. Additionally, autophagy activation was confirmed via atg6 (Beclin-1 ortholog in yeast) gene expression, which is known to play a key role in autophagy maturation and to be upregulated in transcriptional levels (Duncan et al., 2018). In this context, we evaluated Atg6 mRNA levels as shown in Figure 1C, which were increased twofold and threefold in response to camphor and arsenic exposures at 0.4 mg/mL and 0.1 mM concentrations. On the other hand, atg8 mRNA levels were not altered significantly in any of experimental groups (Figure 1D). 

**Table:** Gene-specific primers used in RT-PCR reactions in this study.

Atg6	Forward: 5’ TCGCGATCCTAATGGAAATC 3’Reverse: 5’ AGGGTAAAACCGCTGAAGGT 3’	Tm: 60.0 °CTm: 60.0 °C	Product size:173 bp
Atg8	Forward: 5’ TCTCAAAGAATCCGGGAAAA 3’Reverse: 5’ GGGGGCAAGATTTCATCAAT 3’	Tm: 59.6 °CTm: 61.0 °C	Product size:206 bp
Act1	Forward: 5’ TGTATTCCCCTCGATTGTCGG 3’Reverse: 5’ CACGCTTGCTTTGAGCTTCAT 3’	Tm: 59.6 °C Tm: 60.1 °C	Product size:101 bp

### 3.2. Growth inhibition is substantially dependent on autophagy at low doses of camphor and arsenic

**Figure 2:**
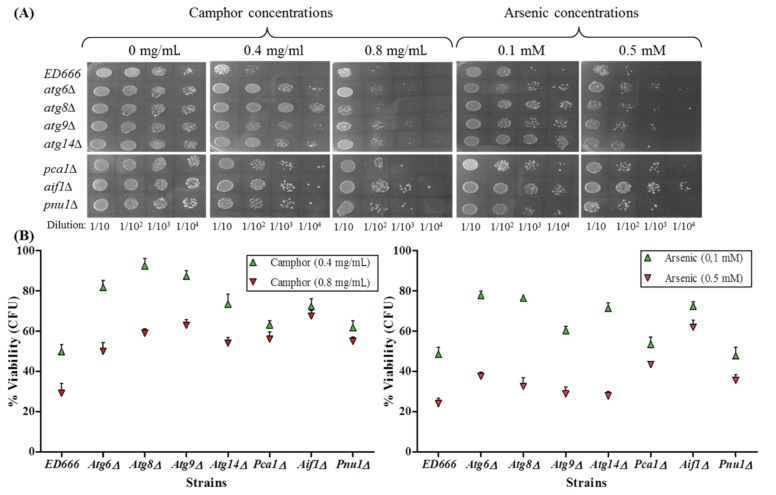
Cell viability of autophagy- and apoptosis-deficient cells. Cell viability was assessed by spot assay (A) and colony-forming assay (B) in comparison to 0.05% ethanol (solvent) control (0 mg/mL refers to solvent control). Values are presented as mean ± SEM. Calculations were made from at least 3 independent biological replicates (n = 3). Serial fivefold dilutions of yeast cells were spotted on YEA plates including gradually increasing concentrations of camphor (0–0.8 mg/mL) and 0.1–0.5 mM arsenic(III). Colonies were counted after incubation at 30 °C for 3 days.

Cellular growth and viability in yeast are dependent on intrinsic and extrinsic factors, including pH, temperature, and environmental and chemical stress, and the resulting cellular and physiological consequences are particularly capable of activating conserved programmed cell death (PCD) subroutines. Autophagy is known as one of the PCD types according to many researchers, while others believe that this type of cell fate should not be classified as cell death machinery. In any case, as a result, growth inhibition and the underlying mechanisms in response to stress conditions should be investigated and explained. We designed an experimental plan including spot assays and colony-forming assays performed with autophagy mutants *atg6∆*,* atg8∆*,* atg9∆*,**and* atg14∆*, which are known to play roles in the activation and regulation of autophagy (Mukaiyama et al., 2009). As demonstrated in Figure 2A, the spot assay showed a dramatic inhibition in wild-type cells at 0.4 mg/mL camphor concentration, whereas *atg* mutants were almost not affected at this concentration. In contrast, control and mutant cells were completely dead at 0.8 mg/mL camphor concentration. Arsenic exposure showed similar results (see Figure 2A). While 0.1 mM arsenic caused a significant inhibition in wild-type cells, autophagy-deficient cells were not affected and showed regular spots similar to the solvent control group. In the high-dose arsenic group (0.5 mM), nearly 50% of mutant cells, as well as wild-type cells, were dead, which was very similar to the camphor-dependent cell death demonstrated at 0.8 mg/mL concentration. Atg8-deficient cells showed a strong resistance to 0.4 mg/mL camphor, but this effect was not shown at high doses of camphor or arsenic (see Figure 2B), suggesting that Atg8 is responsible for efficaciously activating autophagy at low doses of camphor and arsenic.

### 3.3. Aif1 is potentially responsible for apoptosis at high doses of camphor and arsenic

**Figure 3:**
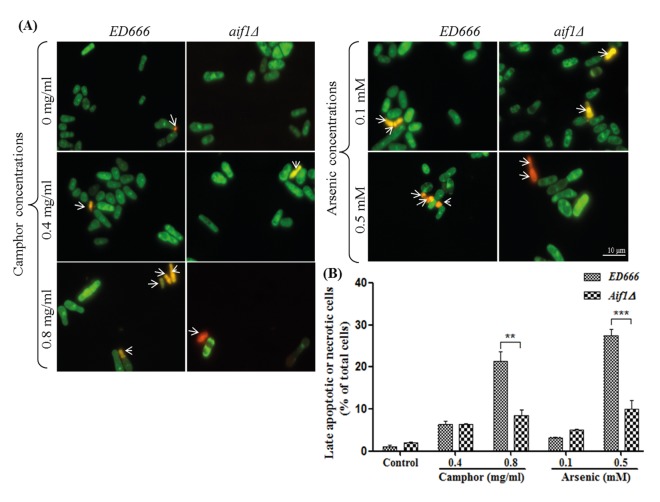
Apoptosis of S. pombe cells. Viable and late apoptotic or necrotic cells were visualized using fluorescent microscope after exposure to 0, 0.4, and 0.8 mg/mL camphor and 0.1 and 0.5 mM arsenic(III) solutions (A). Percentages (B) of late apoptotic or necrotic wild-type (ED666) and aif1Δ cells after exposure to 0–0.8 mg/mL camphor and 0.1–0.5 mM arsenic solutions are shown in corresponding graphics. Values are presented as mean ± SEM. Significantly different values are indicated by asterisks (two-way ANOVA, **P < 0.01, ***P < 0.001). At least 200 cells were counted in each biological replicate (n = 3). Arrows: Late apoptotic or necrotic cells.

When we spotted apoptosis-deficient cells against 0.4 mg/mL camphor exposure, there was a dramatic inhibition in all mutants (*pca1∆*, *aif1∆*, and *pnu1∆*), as shown in Figure 2A, whereas there was a strong correlation between high-dose camphor exposure (0.8 mg/mL) and drastic cell death. However, *aif∆* cells were resistant (or a mild effect was seen) to high-dose camphor exposure, indicating a potential contribution to camphor-dependent apoptosis. A similar cell death pattern was found at low- and high-dose arsenic concentrations (see Figure 2A, right side) proving the key role of Aif1 in camphor- and arsenic-dependent apoptotic cell death. Spot assay results were confirmed by colony-forming assay and quantitative analysis was performed (Figure 2B). The viability of wild-type cells decreased to 50% and 30% at 0.4 mg/mL and 0.8 mg/mL camphor concentrations, which was very similar to that of arsenic at 0.1 mM and 0.5 mM concentrations. Camphor concentration of 0.8 mg/mL caused a dramatic decrease in *pca1∆* and *pnu1∆* cells (viability was 57% and 55%, respectively), but *aif1∆* cells were not strongly affected (viability was 70%), confirming the spot assay results demonstrated in Figure 2A. Similar results were shown in the high-dose arsenic group (43%, 72%, and 31% in *pca1∆*, *aif1∆*, and *pnu1∆* cells, respectively). Apoptosis was confirmed with AO/EB dual staining assay (see Figure 3A), which showed variation in apoptotic cell death ratios. As demonstrated in Figure 3B, results were the same and calculated as 7% in wild-type and *aif1∆* cells at 0.4 mg/mL camphor, whereas the ratios increased 22% in wild-type cells at 0.8 mg/mL camphor (P**< 0.01) without a significant alteration in *aif1∆* cells. Arsenic exposure caused similar ratios at low and high doses (28% and 10% in wild-type and *aif1∆ *cells, P**< 0.001). The resistance of *aif1*-deficient cells to apoptotic cell death in response to high-dose camphor and arsenic shows that Aif1 mediates camphor- and arsenic-induced apoptosis in *S. pombe*. 

## 4. Discussion

As we previously reported, high-dose camphor exposure (more than 0.8 mg/mL) significantly alters ROS levels and oxidative status, mitochondrial metabolism and membrane potential, cell viability, and nuclear morphology of *S. pombe* cells (Agus et al., 2019). In this study, we evaluated the effects of low-dose camphor exposure, in comparison to high-dose camphor exposure, on cell growth and viability of *S. pombe* cells. We were interested in understanding the cause of cellular growth inhibition that could be related to one of the programmed cell death subroutines. We visualized GFP-Atg8 dots, which surround autophagosomes after proteolytic cleavage of several amino acid residues in the C-term of Atg8, known as an end-point marker for autophagy activation (Klionsky, 2011; Torggler et al., 2017). We also evaluated Atg6 (Beclin-1 ortholog) and Atg8 mRNA levels in response to camphor and arsenic exposure. Atg6 mRNA levels increased two- to threefold in both the low-dose camphor and arsenic groups, although Atg8 mRNA levels did not alter in any experimental group. Our results showed that low-dose camphor (0.4 mg/mL) caused a strong autophagy activation (see Figures 1A and 1B), which is similar to low-dose arsenic exposure, known to induce autophagy in human cancer cell lines, i.e. BEAS-2B cells (Zhang et al., 2012) and U-373-MG cells (Kanzawa et al., 2003), or in mouse liver (Zeinvand-Lorestani et al., 2018).

When camphor and arsenic doses increased to higher levels (two- to fivefold), cells in both experimental groups showed a significant decrease in the number of GFP-Atg8 dots, although cell viability decreased to its lowest levels and a strong cell death signal was activated, as shown in Figures 2 and 3. Spot assay, colony-forming assay, and AO/EB staining helped us to understand whether autophagy or apoptosis (or both) is responsible for growth inhibition and cell death. Autophagy-deficient cells (*atg* mutants) did not show any growth defect in the low-dose experimental group, whereas viability of wild-type cells (*ED666*) was diminished to almost its lowest levels, which proves that autophagy is the responsible mechanism for reduction in viability in response to low-dose camphor and arsenic. In contrast, when high-dose camphor and arsenic were used, viability markedly decreased in all strains, including autophagy mutants. We thought that the underlying mechanism for the strong inhibitory effect of high-dose exposure was related to another type of cell death, the apoptotic subroutine of PCD. In this context, we evaluated viability of apoptosis-deficient cells under high-dose camphor and arsenic exposure. While low-dose exposure caused a dramatic inhibition in apoptosis-deficient cells along with wild-type cells, high-dose exposure caused drastic cell death in all mutants except *aif1∆* cells (or a mild effect was seen), which demonstrates to us that Aif1 is the responsible protein for high-dose camphor- and arsenic-induced cell death. In addition, AO/EB staining confirmed our results (see Figure 3). Given that arsenic(III) oxide activates apoptosis via yeast metacaspase Yca1 (*S. cerevisiae* ortholog for Pca1) and mitochondrial disruption (Du et al., 2007), and there is no literature on camphor-induced apoptosis or autophagy and the responsible protein in yeast, this study is the first to declare that Aif1 is responsible for arsenic- and camphor-induced apoptosis in the fission yeast *S. pombe*.

In mammalian cells, phosphorylation of serine and threonine residues (Ser70 and Thr119) promotes direct binding of Bcl-2 to Beclin-1, causing a crosstalk between autophagy and apoptosis, which leads to inhibition of autophagy (Li et al., 2016; Chen et al., 2019). Another possible crosstalk followed by inhibition of autophagic flux is driven by p53, which downregulates LC3 (Li et al., 2016). Although possible crosstalk mechanisms are understood in mammalian cells, the crosstalk of yeast autophagy and apoptosis is under debate and should be unraveled, as p53, Bcl-2 and other regulatory components of apoptosis are absent in yeast. However, in this study, Atg6, which is orthologous to Beclin-1, was transcriptionally downregulated in response to high-dose camphor exposure (0.8 mg/mL), whereas low-dose camphor (0.4 mg/mL) transcriptionally activated Atg6. On the other hand, similar to mammalians, autophagy proteins evolved in the fission yeast (*S. pombe*), which modulate autophagosome formation leading to autophagic cell death. When we used autophagy-deficient (*atg* mutants) cells, low-dose camphor (0.4 mg/mL) did not cause cell death, whereas *atg* mutants and wild-type cells, except apoptosis-deficient cells (*aif1* mutant), completely died at a high dose of camphor (0.8 mg/mL). Moreover, the number of GFP-Atg8 dots per cell decreased twofold in high-dose camphor (see Figure 1). This study experimentally demonstrates that one type of cell death machinery, autophagy, is suppressed when the other type of cell death (apoptosis) is activated. These findings indicate possible crosstalk between autophagy and apoptosis when cells are exposed to a concentration gradient of camphor.

Camphor was shown to cause growth inhibition and cell death in human colon cancer cell line HCT-116 at a lower dose (0.15 mg/mL) (Itani et al., 2008) than that of our study. The reason for the difference in camphor doses is possibly limited permeability of the yeast cell wall, which may reduce the uptake of camphor (Klis et al., 2002), although camphor and other lipophilic monoterpenes are capable of passing through the cell membrane (Gohel and Nagori, 2009; Aydin et al., 2013). Lipid peroxidation, mitochondrial impairment, increased ROS levels, and oxidative stress were declared as the reasons for the antifungal action of monoterpenes (Mutoh et al., 2011; Marei et al., 2012) and camphor-induced cell death, as shown by our previous study (Agus et al., 2019). Molecular, metabolic, and cytotoxic effects of potential anticancer drugs, along with cell death mechanisms, can be understood using fission yeast (*S. pombe*). This study showed that camphor induces 2 types of intrinsic cell death mechanisms, autophagy and apoptosis, in a dose-dependent manner. We used fission yeast as a screen for drug candidates in this study along with their mechanisms of action, with which we can analyze the gene-function relation for *aif1*. Among other cellular targets for cell death, *aif1* was shown to be a strong target for camphor, which can be adapted to mammalian cancer cell lines carrying the orthologous *aif1* gene. Considering these findings, caspase-independent cell death (*aif1*-dependent in this case) activated after camphor exposure can be further investigated in mammalian cancer cells. 

In conclusion, cellular growth and viability markedly decreased after camphor exposure. Autophagic growth inhibition induced by low-dose camphor and arsenic was switched to Aif1-dependent apoptotic cell death in excessive doses. This study demonstrated the potential toxic and inhibitory effects of camphor and other mechanisms in the unicellular model organism *S. pombe*. However, this warrants further study. The literature is still lacking sufficient in vivo experimental data.

**Acknowledgment/Disclaimers/Conflict of interest**

This work was supported by TÜBİTAK (the Scientific and Technological Research Council of Turkey), 2209-A Undergraduate Research Support Program [1919B011701974], and by the board of trustees of İstanbul Yeni Yüzyıl University. We wish to thank Bedia Palabıyık and YGRC/NBRP Japan for providing the *S. pombe *strains, Emre Yörük for consumables and chemicals, and Cenk Kig for sharing his advice and experiences*. *There are no conflicts of interest to declare**.**
